# The impact of age at presentation on lung cancer staging

**DOI:** 10.7196/AJTCCM.2020.v26i2.045

**Published:** 2020-06-15

**Authors:** N A Mhlana, C F N Koegelenberg

**Affiliations:** Division of Pulmonology, Faculty of Medicine and Health Sciences, Stellenbosch University and Tygerberg Hospital, Cape Town, South Africa

**Keywords:** Lung Cancer, presenting stage, age, NSCLC, malignancy, squamous cell carcinoma, large-cell carcinoma

## Abstract

**Background:**

Primary lung cancer is one of the most common causes of cancer and of death due to cancer worldwide. The tumour node metastases staging for non-small-cell lung cancer (NSCLC) helps to prognosticate and plan for treatment. Most patients have advanced disease at the time of diagnosis. Primary lung malignancy was previously diagnosed mostly in older individuals.

**Objectives:**

The primary aim of this study was to determine whether younger age at presentation is a risk factor for more advanced disease. We defined younger age as <45 years.

**Methods:**

This was a retrospective analytical study covering 5.5 years. The information was obtained from the lung cancer registry of all patients presented at our Division of Pulmonology weekly combined oncology meeting.

**Results:**

A total of 52 of 1 083 patients with lung malignancy were <45 years, and 48 of these had NSCLC. Adenocarcinoma was the predominant type (48%), followed by squamous cell carcinoma (27%), NSCLC not otherwise specified (NOS; 21%) and large-cell carcinoma (4%). Overall, the majority of patients (98%) had advanced disease at presentation. However, there was no statistical difference compared with presenting stage in older patients (odds ratio 0.25, 95% confidence interval (CI) 0.034 - 1.874 and risk ratio 0.27 (95% CI (0.038 - 1.900)).

**Conclusion:**

Primary lung malignancy remains a disease of the elderly. This study demonstrated that NSCLC tends to present in advanced stages in younger patients, although the difference was not statistically significant.

## Background

Primary lung cancer is one of the most common causes of cancer, and the most common cause of death due to cancer worldwide. The total number of cases worldwide is estimated to be 1.8 million, and more than half of cases come from developing countries.^[1,2]^ In South Africa (SA), it is a frequently diagnosed disease and remains a growing health problem in both men and women.^[3,4]^ The risk factors for primary lung cancer include cigarette smoking, genetic predisposition, environmental/occupational exposure, underlying chronic lung disease such as lung fibrosis, and chronic obstructive pulmonary disease. HIV infection and the presence of malignancies elsewhere are added risk factors.^[2]^ The two broad categories of lung cancer are non-small-cell lung cancer (NSCLC) and small-cell (SC) lung cancer. NSCLC is the more common type, and is further classified into adenocarcinoma, squamous cell carcinoma, large-cell carcinoma, and NSCLC not otherwise specified (NOS). The tumour node metastases (TNM) system is used to stage NSCLC, in order to assess severity, prognosticate and explore treatment options. The staging is graded from stage I to IVb. Stages I - IIIa are regarded as early stages of the disease, and treatment is often surgical resection performed with curative intent. Stages IIIb - IV are more advanced stages with poorer prognosis, and palliation or supportive care is the only treatment option that can be offered.^[6]^

Primary lung malignancy was previously regarded as a disease of the elderly, mostly affecting patients between 50 and 80 years of age, and rarely seen in patients <45 years old.^[2,9]^ Therefore the majority of research on primary lung cancer, in terms of diagnostics, prognostication and treatment, has so far focused on the older age group. Whether younger age (<45 years) at presentation is a significant poor prognostic factor remains to be explored. Previous research to compare the extent of the disease between younger and older patients (>45 years) at presentation, and the influence of this on prognosis, is, to the best of our knowledge, limited. A comparison study by Yazgan *et al*. ^[9]^ evaluated the effect of age on survival after lung cancer surgery. Their younger population was <45 years old, and those >70 years were classified as elderly. The younger patients presented with more advanced stage (III - IV), but had better survival compared with the elderly group owing to other comorbidities in this group.

A study by Tas *et al*. ^[2]^ investigated the clinical importance of age at presentation in lung cancer patients. The median (range) age in the study was 59 (35 - 88) years. They concluded that elderly patients had a poorer prognosis compared with the younger population group. They further suggested that possible reasons might be that older patients tend to have multiple comorbidities, poor performance status, and generally present with more advanced disease. Another multicentre study by Bourke *et al*. ^[6]^ looking at patients <45 years of age with lung cancer showed that these patients presented predominantly with stage I or II disease and were likely to receive surgical treatment with curative intent. Other studies suggested that the reason for a poorer prognosis in younger patients was that they tended to present with the adenocarcinoma histological type, which is considered to be a more aggressive type of NSCLC.^[2,7,10,11]^

The primary aim of the present study was to investigate whether younger patients (≤45 years) with lung cancer presenting to Tygerberg Hospital’s Division of Pulmonology present with more virulent disease as determined by TNM staging, compared with older patients (>45 years).

## Methods

### Study design

This was a retrospective analytical study over a period of 5.5 years from June 2012 to December 2017, reviewing all patients diagnosed with lung cancer at Tygerberg Hospital’s Division of Pulmonology. The lung cancer registry of all patients presented at the division’s weekly combined oncology meeting was used to collect data. All adult patients (>18 years) were included. These were split into two cohorts of younger (≤45 years) and older (>45 years) patients for comparison. All patients had a computed tomography scan to determine TNM staging. Histological diagnosis was confirmed and validated using the laboratory report on the local intranet (hospital network).

The study was conducted in accordance with the Belmont Report guidelines for the protection of human research subjects. All data were collected and stored in a password-protected database with access limited to the investigators. The application included a waiver of consent due to the retrospective nature and anonymity of the study design.

### Statistical analysis

Descriptive statistics and χ^2^ comparisons of proportional data were performed. A p-value <0.05 in a two-tailed test of proportions was considered significant. Unless otherwise stated, data are displayed as means and standard deviations (SDs). Vassarstats (Richard Lowry, USA) was used for statistical analysis.

## Results

In this retrospective study, we included a total of 1 083 patients from the lung cancer registry for analysis. Fifty-two patients <45 years old were included, and 1 031 older patients for comparison. In the younger age group there were 34 males, with a mean (SD) age of 40.2 (4.3) years. A total of 48 (n=48/52; 92.3%) patients in this age group had confirmed NSCLC and were analysed for the purpose of this study. The majority (48%) had adenocarcinoma histological type. The remaining histological types were squamous cell carcinoma (27%), NSCLC NOS (21%) and large-cell carcinoma (4%).

The older group included 653 males, and the mean (SD) age was 61.6 (9.0). Of the 1 031 patients in this age group, 895 (87%) were diagnosed with NSCLC. In terms of histological type predominance, the trend was similar to the younger age group. Adenocarcinoma was the most common and accounted for 54% of the cases, with 33% squamous cell carcinoma, 9% NSCLC NOS and 4% large-cell carcinoma. A summary of these findings is represented in (Tables 1 and 2).

We also noticed that the majority of patients in both age groups presented with advanced stage disease. Of the 48 younger patients, 98% (n=47) presented with stages IIIb - IVb. Similarly, in the older age group, the majority (92%) presented in advanced stages. The odds ratio was 0.25 (95% confidence interval (CI) 0.03 - 1.87). The risk ratio was 0.27 (95% CI 0.04 - 1.90) with a p-value of 0.17. (Tables 3 and 4) present a summary of these findings.

## Discussion

There is a paucity of studies looking at the extent and aggressiveness of primary lung cancer in younger people. The TNM staging system is used to determine the extent of the disease and to prognosticate. Early stages of the disease carry a favorable prognosis, while advanced stages represent a poorer prognosis. Tas *et al*. ^[2]^ concluded that older age was a significant poor prognostic factor in patients with primary lung cancer. They further explored possible reasons for this, which included the fact that older patients tend to have multiple comorbidities and frailty. Few studies have explored possible reasons for a poorer prognosis in younger patients, but have suggested that the predominance of adenocarcinoma histological type in this age group might be contributory, as this type was thought to be more aggressive than other types of NSCLC.^[6,10]^

In our study, the older cohort was much larger than the younger. This confirms what has already been demonstrated: that there is a higher prevalence of primary lung cancer in older people than younger. A study by Subramanian *et al*. ^[7]^ also suggested that elderly people presented more commonly with squamous cell type than younger patients, and the authors thought it carried a poorer prognosis. In our study, adenocarcinoma was the most common histological type in both age groups, accounting for 48% in younger patients and 54% in older patients. Squamous cell carcinoma was the second most common, followed by NSCLC NOS, and large-cell type as the least common in both age groups (Tables 1 and 2).

In terms of TNM staging, our results demonstrate a similar trend in both age groups, whereby the majority of patients presented in advanced stages of the disease (Tables 3 and 4). Younger patients had a higher proportion of advanced stage disease than older patients. However, this difference in proportions was not statistically significant.

Although lung cancer occurs predominantly in older patients, it is imperative to suspect it in younger patients who present with suggestive symptoms and signs. Previous studies have suggested that the reason for the rate of advanced disease in younger patients is low index of suspicion, and the common practice of trial of treatment for common respiratory tract infections such as pulmonary tuberculosis (TB) and community-acquired pneumonia, while the underlying (unsuspected) malignancy worsens. The index of suspicion for malignancy only rises if patients fail to improve on antibiotics or antiTB treatment. A proportion of patients in the younger age group in our study might have undergone this kind of management strategy, especially given the high incidence of pulmonary TB in SA. A study by Bourke *et al*. ^[6]^ found that younger patients presented with early-stage disease compared with older patients, and received curative treatment, but it was conducted in a developed country where the prevalence of infectious disease is lower and the index of suspicion for malignancy higher. Our study found NSCLC diagnosed in advanced stages in both younger and older people, suggesting that it should be placed higher in differential diagnoses in younger patients with risk factors and suggestive symptoms and signs.

### Study strengths and limitations

The strength of our study is that owing to its retrospective nature, we were able to include a large sample for analysis. A study limitation is that we may not have included all young patients with primary lung cancer, as some might have been too ill for diagnosis at presentation, perhaps due to misdiagnosis initially, and died shortly after.

## Conclusion

Our study did not demonstrate a statistically significant difference in terms of disease stage at presentation between younger and older patients, although younger patients had a higher proportion of advanced stage disease than older patients. The prevalence of lung cancer is noticeable in patients <45 years of age, therefore, diagnostic investigations should be performed early to exclude it. Delayed diagnosis due to low index of suspicion of lung malignancy in younger patients is likely the predominant reason for advanced disease stages at presentation.

## Figures and Tables

**Table:** Table 1. Histological types in patients ≤45 years (*N*=48)

Histological type	*n *(%)
Adenocarcinoma	23 (48)
Squamous cell carcinoma	13 (27)
NSCLC NOS	10 (21)
Large cell	2 (4)

NSCLC NOS = non-small-cell lung cancer not otherwise specified.

**Table:** Table 2. Histological types in patients >45 years (*N*=895)

Histological type	*n *(%)
Adenocarcinoma	480 (54)
Squamous cell carcinoma	301 (33)
NSCLC NOS	81 (9)
Large cell	33 (4)

NSCLC NOS = non-small-cell lung cancer not otherwise specified.

**Table:** Table 3. TNM stages in patients ≤45 years (*N*=48)

Stage	*n *(%)
I - IIIa (early stage)	1 (2)
IIIb - IVb (advanced stage)	47 (98)

TNM = tumour node metastasis.

**Table:** Table 4. % of TNM stages in patients >45 years (*N*=895)

Stage	*n *(%)
I - IIIa (early stage)	69 (8)
IIIb - IVb (advanced stage)	826 (92)

TNM = tumour node metastasis.
